# A tightly controlled fMRI dataset for receptive field mapping in human visual cortex

**DOI:** 10.1016/j.dib.2023.109018

**Published:** 2023-03-01

**Authors:** Joram Soch, Kai Görgen, Jakob Heinzle, John-Dylan Haynes

**Affiliations:** a Berlin Center for Advanced Neuroimaging, Charité – Universitätsmedizin Berlin, Corporate Member of Freie Universität Berlin, Humboldt-Universität zu Berlin, and Berlin Institute of Health, Sauerbruchweg 4, 10117 Berlin, Germany; b Berlin Center for Computational Neuroscience, Philippstraße 13, Haus 6, 10115 Berlin, Germany; c German Center for Neurodegenerative Diseases, Von-Siebold-Straße 3A, 37075 Göttingen, Germany; d Translational Neuromodeling Unit, Institute for Biomedical Engineering, University of Zurich and ETH Zurich, Wilfriedstrasse 6, 8032 Zürich, Switzerland; e Berlin School of Mind and Brain, Humboldt University of Berlin, Luisenstraße 56, Haus 1, 10117 Berlin, Germany; f Department of Psychology, Humboldt University of Berlin, Germany, Wolfgang Köhler-Haus, Rudower Chaussee 18, 12489 Berlin; g EXC Science of Intelligence, Technical University of Berlin, Marchstr. 23, 10587 Berlin, Germany; h CRC Volition and Cognitive Control, Technical University of Dresden, Zellescher Weg 17, 01069 Dresden, Germany

**Keywords:** Magnetic resonance imaging, Functional MRI, Visual perception, Retinotopic mapping, Angular direction, Eccentricity, Flickering checkerboard, Continuous stimulation

## Abstract

Four right-handed, healthy subjects participated in a visual stimulation experiment. Subjects were viewing a dartboard-shaped flickering checkerboard stimulus, divided into 4 rings and 12 segments, defining 48 sectors in the visual field. Local contrast in each sector was continuously varying across four levels and updated every 3 s. To maintain fixation, subjects had to respond to a stimulus at the center of the visual field.

During the entire experiment, in which subjects performed 8 runs, each consisting of 100 trials, brain activity was measured with functional magnetic resonance imaging (MRI). Using a 3-T Siemens Trio MRI scanner, 220 echo-planar images were acquired in each run, with a repetition time of 1.5 s and voxel size of 3 x 3 x 3 mm.

The dataset is publicly available from OpenNeuro and additionally includes region of interest maps for visual areas V1 to V4, left and right, obtained from another retinotopic mapping experiment. As such, the dataset allows for accurate mapping of receptive fields and their properties across several stages of human visual cortex.

## Graphical abstract

**Figure:**
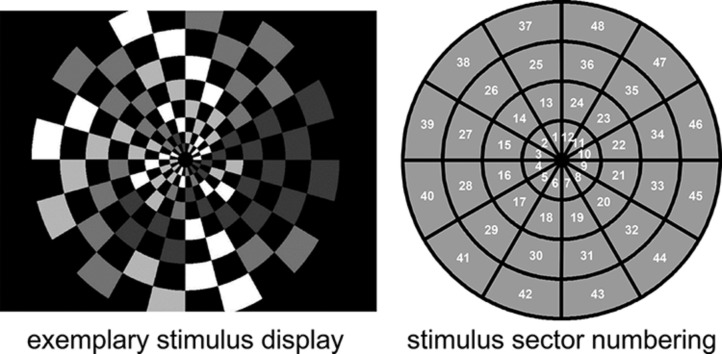

**Specifications Table**

**Table:** 

Subject	Biological Sciences → Neuroscience → Sensory Systems
Specific subject area	investigation of brain activity underlying human visual perception, measured with functional magnetic resonance imaging
Type of data	structural brain images (NIfTI files [.nii]) functional brain images (NIfTI files [.nii]) experimental design information (tab-separated values [.tsv]) experiment meta-data (Java Script objection notation [.json])
How the data were acquired	All data were collected on a 3-T Siemens Trio MRI scanner with a 12-channel head coil. For functional MRI, 220 T2*-weighted, gradient-echo EPIs were acquired in each run at a repetition time TR = 1.5 s, echo time TE = 30 ms, flip angle α = 90° and in 25 slices (slice thickness: 2 mm [+1 mm gap]; matrix size: 64 x 64; field of view: 192 x 192 mm), resulting in a voxel size of 3 x 3 x 3 mm. For structural MRI, a single T1-weighted MPRAGE image was acquired at a repetition time TR = 1.9 s, echo time TE = 2.52 ms, flip angle α = 90° and in 192 slices (matrix size: 256 x 256) with a voxel size of 1 x 1 x 1 mm.
Data format	Raw Analyzed
Description of data collection	Subjects were looking at a visual stimulus that was divided into 48 sectors to control stimulation at different locations in the visual field. Across trials, each sector changed its illumination intensity to one out of four levels. The duration of one trial was 3 seconds and there was no inter-stimulus interval. A control task was implemented to direct subjects’ attention to the center of the stimulus at all times.
Data source location	Institution: Charité – Universitätsmedizin Berlin, Campus Benjamin Franklin (Hindenburgdamm 30, 12203 Berlin) City and country: Berlin, Germany Latitute and longitude: 52.44163 N, 13.32045 E
Data accessibility	Repository name: OpenNeuro Data identification number: ds002013 Digital object identifier: 10.18112/openneuro.ds002013.v1.0.3 Direct URL to data: https://openneuro.org/datasets/ds002013/ License: CC0 Instructions for access: see https://openneuro.org/datasets/ds002013/versions/1.0.3/download
Related research article	J. Heinzle, T. Kahnt, J.-D. Haynes, Topographically specific functional connectivity between visual field maps in the human brain, NeuroImage. 56 (2011) 1426-1436. https://doi.org/10.1016/j.neuroimage.2011.02.077

## Value of the Data

• Neuronal cells in visual cortex are characterized by receptive fields (RF) which means that individual neurons respond to stimulation in specific parts of the visual field. This concept has been extended to neuronal populations (pRFs) and voxels (vRFs) sampled with functional magnetic resonance imaging (fMRI). The present dataset allows to comprehensively map visual space to visual cortex by offering a tightly controlled visual stimulus and many data points in each individual subject.
• The dataset can be useful for three groups of people: Visual neuroscientists can investigate the data to learn more about the organization of early and later visual cortices [1]. Methods developers can employ the dataset for empirical validation of novel approaches for functional magnetic resonance imaging (fMRI) data analysis [2]. Finally, university teachers can use the data to teach students how to infer on receptive fields using fMRI data and to introduce them to the architecture of visual cortex.
• The human visual system is one of the best-studied sensory systems in all of neuroscience. The concept of pRFs generalizes the idea of receptive fields to large groups of neurons which can be efficiently investigated with fMRI. Beyond the already established knowledge about the human visual system, the present dataset allows to research (i) higher-order interactions of receptive fields, i.e. integration of early visual representations in later stages of visual cortex; and (ii) sequence effects in visual perception, i.e. the influence of previous stimulation on the cortical response to current stimulation.

## 1 Objective

Human visual cortex is organized in a retinotopic fashion [3,4]. This means that distinct parts of cerebral cortex process information about distinct parts of the visual field and neighboring places in the visual field are represented in neighboring areas of visual cortex. To optimally infer on this world-brain relationship, a tightly controlled visual stimulation experiment was devised. In particular, a circular stimulus was used and partitioned into individual parts based on polar coordinates – in order to account for the known angle-eccentricity representation in visual cortex. Moreover, stimulation in each part of the stimulus was a flickering checkerboard pattern – in order to avoid known saturation and adaptation effects in neuronal activity. The data were generated for a previous article on the investigation of cortico-cortical receptive fields [1] and are here provided in order to allow for the full range of possible analyses.

## 2 Data Description

The dataset is organized in the brain imaging data structure (BIDS) file format [5] for magnetic resonance imaging (MRI) and available from OpenNeuro [6]. As such, it contains the following Java Script object notation (JSON) and tab-separated value (TSV) files in the main directory:

• “dataset_description.json”: a JSON file with basic information about the dataset
• “participants.json”: a JSON file containing subject-wise information (age, gender etc.)
• “participants.tsv”: a TSV file specifying participant IDs and other participant information
• “task-CircRun_bold.json”: a JSON file detailing functional MRI acquisition parameters
• “task-CircRun_events.json”: a JSON file explaining trial-wise covariates
• “README”: a plain text file describing files contained in the dataset

Moreover, there is one sub-folder per subject in the main directory (e.g. “sub-AAA02”) which contains the following files (X = subject ID, e.g. “AAA02”):

• “data2BIDS_*_X.mat”: MAT-files specifying batch editor jobs for Statistical Parametric Mapping, Version 12 (SPM12) used for converting the data to the BIDS format
• “preproc_X.mat” a MAT-file specifying a batch editor job for preprocessing the data in SPM12 equivalent to the preprocessing procedure used in related work [2]

In each subject folder, there is also a sub-directory called “anat” containing the following files:

• “sub-X_T1w.json”: a JSON file detailing structural MRI acquisition parameters
• “sub-X_T1w.nii”: a skull-stripped, but otherwise unprocessed T1-weighted MPRAGE image of the subject in NIfTI format
• “sub-X_roi-Y-Z.nii”: a NIfTI file specifying a binary mask for region of interest (ROI) analysis in preprocessed image space, when using the preprocessing routines mentioned above; X is the subject ID, Y gives the brain region (V1, V2a, V2b, V3a, V3b, V4) and Z gives the brain hemisphere (left or right); an exemplary filename would thus be “sub-AAA02_roi-V4-left.nii”

In each subject folder, there is also a sub-directory called “func” containing the following files:

• “sub-X_task-CircRun_run-YY_bold.nii”: a 4D NIfTI file storing the unprocessed functional MRI scans from a single run obtained during the experiment of interest; X is the subject ID and Y corresponds to the run number (1 ≤ Y ≤ 8); an exemplary filename would thus be “sub-AAA02_task-CircRun_run-01_bold.nii”
• “sub-X_task-CircRun_run-YY_events.tsv”: a TSV file storing onsets, durations and trial-wise modulator values for all trials from a single run
• “sub-X_task-CircRun_run-YY_events.xls”: the same information, stored in tabular format as a Microsoft Office Excel spreadsheet
• “sub-X_task-CircRun_run-YY_events.mat”: the same information, stored in SPM12’s “names, onsets, durations” format [7]

Note that the following files are not required per BIDS standard and merely exist for convenience of the dataset consumers:

• “sub-X/data2BIDS_*_X.mat”
• “sub-X/preproc_X.mat”
• “sub-X/anat/sub-X_roi-Y-Z.nii”
• “sub-X/func/sub-X_task-CircRun_run-YY_events.xls”
• “sub-X/func/sub-X_task-CircRun_run-YY_events.mat”

## 3 Experimental Design, Materials and Methods

### 3.1 Participants

Eight right-handed, healthy subjects participated in the visual stimulation experiment. All subjects had normal or corrected-to-normal vision and gave written informed consent to participate in the fMRI experiment. From these eight subjects, we provide the data of four subjects (3 male, 1 female, age range 24-28 yrs; see “participants.tsv” in “Data description”) who also took part in a retinotopic mapping experiment that was used for generating region of interest (ROI) images which are included in the dataset.

### 3.2 Experimental design

In both experiments (visual stimulation and retinotopic mapping), subjects were presented with a high-contrast, flickering circular checkerboard that was subdivided into 48 sectors (see [2], Fig. 6) which changed their local contrast independently over time (see [1], Fig. 2). To avoid spurious order effects, time series of local contrast were randomly generated using M-sequences [8].

Intensity levels were logarithmically spaced between 0.1 and 1

$$x\in \left\{10^{-1},\;10^{-2/3},\;10^{-1/3},\;10^{0}\right\}=\left\{0.1,\;0.215,\;0.464,\;1\right\}$$

and are linearly reported as ranging between 0 and 1

$$y=\left(\log_{10}x+1\right)\in \left\{0,\;1/3,\;2/3,\;1\right\}=\left\{0,\;0.333,\;0.667,\;1\right\}$$

in the logfiles (see “events.tsv” in “Data description”).

Intensity levels changed every 3 seconds and there was no inter-trial interval, yielding a continuous visual stimulation experiment. There were 8 runs of continuous stimulation and each run consisted of 100 trials, i.e. changes of local contrast in all sectors.

Visual stimuli were projected onto a translucent screen at the rear of the scanner (screen size: 25° by 20° of visual angle). The visual stimulus comprised a border along the vertical meridian resulting in completely independent visual stimulation in the two hemifields.

During stimulation, subjects performed a fixation task at the center of the visual display. Landolt's C [9] was presented and subjects had to indicate whether it opened to the left or right side. The open and close times were 800 ms each, with a total stimulus duration of T = 1.6 s in order not to interfere with the acquisition TR = 1.5 s (see “fMRI data acquistion”).

### 3.3 fMRI image acquisition

Magnetic resonance imaging (MRI) data were collected on a 3-T Siemens Trio (Erlangen, Germany) with a 12-channel head coil. In each run of the visual stimulation experiment, 220 T2*-weighted, gradient-echo echo-planar images (EPI) were acquired at a repetition time TR = 1.5 s, echo time TE = 30 ms, flip angle α = 90° and in 25 slices (slice thickness: 2 mm [+1 mm gap]; matrix size: 64 x 64; field of view: 192 x 192 mm), yielding a voxel size of 3 x 3 x 3 mm. In each run of the retinotopic mapping experiment, 160 EPIs were acquired at TR = 2 s with 33 slices (all other parameters as above). Finally, for each subject, a T1-weighted magnetization-prepared rapid acquisition gradient-echo (MPRAGE) image was acquired with TR = 1.9 s, TE = 2.52 ms, flip-α = 9° and 192 slices (matrix size: 256 x 256) with a voxel size of 1 x 1 x 1 mm.

### 3.4 Retinotopic mapping acquisition

Retinotopic mapping was implemented via a traveling wave method with a double wedge and an expanding ring stimulus [10,11]. Angular phase values range from 0 to 2π within each hemisphere and are directly proportional to the azimuth spanning the 180° of one visual hemifield. The eccentricity of the expanding rings was chosen so that the eccentricity phase r also ranges from 0 to 2π and is proportional to log(1+ecc), where ecc is the eccentricity in degrees of visual angle. Six runs (4 for visual angle and 2 for eccentricity mapping) comprising 160 images were measured.

### 3.5 Retinotopic mapping analysis

We used FreeSurfer for gray matter segmentation of the anatomical images [12] and mrGray for cortical flattening [13]. Based on this, flattened angular and eccentricity maps were generated using in-house MATLAB scripts. Looking at the angular maps, we defined the borders between early visual areas V1, V2, V3 and V4 by visual inspection. Visual areas V2 and V3 were additionally sub-divided into ventral (V2a, V3a) and dorsal (V2b, V3b) parts. The ROIs for visual areas V1 to V4 were specified based on these borders on the flattened surface, transformed back to three-dimensional anatomical space and then to the functional space of the echo-planar images.

## Ethics Statements

Written informed consent was obtained from all subjects before participating in the experiments. The study was approved by the ethics committee of the University of Leipzig, Germany (protocol number 953) and conducted according to the Declaration of Helsinki [14].

## CRediT Author Statement

**Joram Soch:** Formal analysis, Writing – Original Draft; **Kai Görgen:** Software, Formal analysis, Data curation; **Jakob Heinzle:** Conceptualization, Methodology, Software, Validation, Formal analysis, Investigation, Data curation, Project administration, Writing – review & Editing; **John-Dylan Haynes:** Conceptualization, Methodology, Resources, Supervision, Funding acquisition.

## Declaration of Competing Interest

The authors declare that they have no known competing financial interests or personal relationships that could have appeared to influence the work reported in this paper.

## Data Availability

Visual Receptive Field Mapping Experiment (Original data) (OpenNeuro).
